# Updated evidence-based screening and treatment of osteoporosis in spinal cord injured patients

**Published:** 2012-11

**Authors:** Radin Maheronnaghsh, Ali Yousefian, Vafa Rahimi-Movaghar

**Affiliations:** ^a^Sina Trauma and Surgery Research Center, Tehran University of Medical Sciences, Tehran, Iran.; ^*b*^Research Centre for Neural Repair, University of Tehran, Tehran, Iran.

## Abstract

**Background:**

Spinal cord injury (SCI) can result in a profound reduction in bone mineral density (BMD) and disturbances of the skeletal trabecular microarchitecture. The pathogenesis of osteoporosis after SCI is complex and differs from other forms of this problem. Low-impact fractures in SCI patients usually occur during accidents that should not normally cause fracture. The aim of this study is to review the latest works on evidence-based screening and treatment of osteoporosis in spinal cord injured patients.

**Methods:**

The MedLine andPubMed databases were used as the data source to identify studies from January 1, 1995 to August 1, 2012. Cross referencing of discovered articles was also performed. All of the evidences related to SCI-induced osteoporosis were extracted. Selected ones categorized into two main groups: screening and treatment. We designed search strategies using following keywords in both text word and subject heading forms: “Osteoporosis”, “bone loss”, “spinal cord injury”, “veteran”, “evidence”, and “screening, treatment”. According to the inclusion criteria, 92 studies were included.

**Results:**

Evidences for screening shows different modalities such as Dual energy X-ray absorptiometry (DEXA), Quantitative ultrasound (QUS) and Quantitative computed tomography (QCT), each with its own advantages and disadvantages. Treatment methods include bisphosphonates, vitamin D, Sclerostin monoclonal antibody, standing and walking, exercise, electrical muscle stimulation and cycling, vibration and ultrasound (US).

**Discussion:**

Research in SCI-induced bone loss is in its infancy. The exact pathogenesis of this debilitating complication has not yet been cleared. Furthermore, lack of sufficient evidences and guidelines makes it more difficult to recommend present knowledge for clinical practice purposes.

**Conclusions:**

Considering the high burden of this complication, more studies should be conducted to have a better understanding of the pathophysiology of bone loss in SCI. Future research on new drugs such as Denosumab, an anti-RANKL antibody, would be promising. Finally, health monitoring, accurate diagnosis and targeted treatments should be considered shortly after injury.

**Keywords:**

Osteoporosis, Spinal Cord Injury, Evidence, Prevention, Treatment


					Volume 4, Suppl. 1 Nov 2012
Publisher: Kermanshah University of Medical Sciences
URL: http://www.jivresearch.org
Copyright:
Congress of Iranian Neurosurgeons, 14-16 November 2012, Kermanshah, Iran
Paper No. 6
Updated evidence-based screening and treatment of
osteoporosis in spinal cord injured patients
Radin Maheronnaghsh a
, Ali Yousefian a
, Vafa Rahimi-Movaghar a,b,*
a
Sina Trauma and Surgery Research Center, Tehran University of Medical Sciences, Tehran, Iran.
b
Research Centre for Neural Repair, University of Tehran, Tehran, Iran.
Abstract:
Background: Spinal cord injury (SCI) can result in a profound reduction in bone mineral density (BMD) and
disturbances of the skeletal trabecular microarchitecture. The pathogenesis of osteoporosis after SCI is complex and
differs from other forms of this problem. Low-impact fractures in SCI patients usually occur during accidents that
should not normally cause fracture. The aim of this study is to review the latest works on evidence-based screening
and treatment of osteoporosis in spinal cord injured patients.
Methods: The MedLine and PubMed databases were used as the data source to identify studies from January 1,
1995 to August 1, 2012. Cross referencing of discovered articles was also performed. All of the evidences related
to SCI-induced osteoporosis were extracted. Selected ones categorized into two main groups: screening and
treatment. We designed search strategies using following keywords in both text word and subject heading forms:
"Osteoporosis", "bone loss", "spinal cord injury", "veteran", "evidence", and "screening, treatment". According to the
inclusion criteria, 92 studies were included.
Results: Evidences for screening shows different modalities such as Dual energy X-ray absorptiometry (DEXA),
Quantitative ultrasound (QUS) and Quantitative computed tomography (QCT), each with its own advantages and
disadvantages. Treatment methods include bisphosphonates, vitamin D, Sclerostin monoclonal antibody, standing and
walking, exercise, electrical muscle stimulation and cycling, vibration and ultrasound (US).
Discussion: Research in SCI-induced bone loss is in its infancy. The exact pathogenesis of this debilitating
complication has not yet been cleared. Furthermore, lack of sufficient evidences and guidelines makes it more difficult
to recommend present knowledge for clinical practice purposes.
Conclusions: Considering the high burden of this complication, more studies should be conducted to have a better
understanding of the pathophysiology of bone loss in SCI. Future research on new drugs such as Denosumab, an anti-
RANKL antibody, would be promising. Finally, health monitoring, accurate diagnosis and targeted treatments should
be considered shortly after injury.
Keywords:
Osteoporosis, Spinal Cord Injury, Evidence, Prevention, Treatment
* Corresponding Author at:
Vafa Rahimi-Movaghar: Associate professor of Neurosurgery, Research Deputy, Sina Trauma and Surgery Research Center, Sina Hospital,
Hassan-Abad Square, Imam Khomeini Ave, Tehran University of Medical Sciences, Tehran, Iran. Phone: (+98) 915 342 2682, (+98) 216 6757010,
Fax: (+98) 216 675 7009, Email: v_rahimi@sina.tums.ac.ir , v_rahimi@yahoo.com, (Rahimi-Movaghar V.).

				

